# Development and testing of study tools and methods to examine ethnic bias and clinical decision-making among medical students in New Zealand: The *Bias and Decision-Making in Medicine (BDMM)* study

**DOI:** 10.1186/s12909-016-0701-6

**Published:** 2016-07-11

**Authors:** Ricci Harris, Donna Cormack, Elana Curtis, Rhys Jones, James Stanley, Cameron Lacey

**Affiliations:** Te Kupenga Hauora Māori, Faculty of Medical and Health Sciences, University of Auckland, Private Bag 92019, Auckland, New Zealand; Dean’s Department, University of Otago Wellington, PO Box 7343, Wellington, New Zealand; Māori/Indigenous Health Institute (MIHI), University of Otago Christchurch, PO Box 4345, Christchurch, New Zealand

**Keywords:** New Zealand, Bias, Racial, Ethnic, Medical students, Study development, Pretesting, Pilot, Healthcare

## Abstract

**Background:**

Health provider racial/ethnic bias and its relationship to clinical decision-making is an emerging area of research focus in understanding and addressing ethnic health inequities. Examining potential racial/ethnic bias among medical students may provide important information to inform medical education and training. This paper describes the development, pretesting and piloting of study content, tools and processes for an online study of racial/ethnic bias (comparing Māori and New Zealand European) and clinical decision-making among final year medical students in New Zealand (NZ).

**Methods:**

The study was developed, pretested and piloted using a staged process (eight stages within five phases). Phase 1 included three stages: 1) scoping and conceptual framework development; 2) literature review and identification of potential measures and items; and, 3) development and adaptation of study content. Three main components were identified to assess different aspects of racial/ethnic bias: (1) *implicit* racial/ethnic bias using NZ-specific Implicit Association Tests (IATs); (2) *explicit* racial/ethnic bias using direct questions; and, (3) clinical decision-making, using chronic disease vignettes. Phase 2 (stage 4) comprised expert review and refinement. Formal pretesting (Phase 3) included construct testing using sorting and rating tasks (stage 5) and cognitive interviewing (stage 6). Phase 4 (stage 7) involved content revision and building of the web-based study, followed by pilot testing in Phase 5 (stage 8).

**Results:**

Materials identified for potential inclusion performed well in construct testing among six participants. This assisted in the prioritisation and selection of measures that worked best in the New Zealand context and aligned with constructs of interest. Findings from the cognitive interviewing (nine participants) on the clarity, meaning, and acceptability of measures led to changes in the final wording of items and ordering of questions. Piloting (18 participants) confirmed the overall functionality of the web-based questionnaire, with a few minor revisions made to the final study.

**Conclusions:**

Robust processes are required in the development of study content to assess racial/ethnic bias in order to optimise the validity of specific measures, ensure acceptability and minimise potential problems. This paper has utility for other researchers in this area by informing potential development approaches and identifying possible measurement tools.

**Electronic supplementary material:**

The online version of this article (doi:10.1186/s12909-016-0701-6) contains supplementary material, which is available to authorized users.

## Background

There is well-established evidence of stark, persistent inequities between Māori (the indigenous peoples) and non-indigenous peoples in New Zealand for a range of health measures, including chronic diseases and healthcare [1]. Health professional education and training may play a role in advancing health professional understanding and addressing health inequities between indigenous and non-indigenous populations. In recent years, increased attention is being paid to examining the role of racial/ethnic bias among health professionals [2], within a broader context of research on racism as an underpinning determinant of health and driver of ethnic inequities [3, 4].

Racism can be conceptualised as a societal phenomenon involving ideologies about ‘racial’ and ‘ethnic’ groups grounded in particular histories and socio-political contexts [5]. Within this social system, the categories of ‘race’ and ‘ethnicity’ are constructed, racial hierachies are created and maintained, and manifest as discrimination at personal and structural levels [6, 7]. There are multiple ways by which racism impacts on health both directly and indirectly [4], including the potential impact of racial/ethnic bias amongst healthcare providers [8].

Racial/ethnic bias has been defined as involving “… generally negative feelings and evaluations of individuals because of their group membership (prejudice), overgeneralized beliefs about the characteristics of group members (stereotypes), and inequitable treatment (discrimination)” [9] p201. As van Ryn and colleagues note, healthcare provider bias towards particular racial/ethnic groups occurs within the wider context of racism at a societal level, with clinicians influenced by broader social attitudes and values about race/ethnicity [9]. While people may be aware of their racial/ethnic biases, these can also function at a less conscious or more automatic level [9, 10]. Explicit bias has been described as bias that people are aware of [10] or that is “conscious and intentional” [9], p201, whereas implicit bias is conceptualised as bias that is “unconscious and automatically activated” [9], p201.

Clinician racial/ethnic biases may impact on both the healthcare encounter itself (through influencing provider and patient behaviour or feelings) and decisions about care (by providers and patients) [11, 12]. Physician clinical reasoning and decision-making involves complex cognitive processes that can be further influenced by surrounding environments and individual factors [10, 13]. Physicians often draw on heuristics, or cognitive shortcuts, in decision-making processes particularly under more difficult conditions [13]. While such shortcuts are efficient, they are also prone to error or bias [13]. Racial/ethnic bias is a specific type of acquired or learned bias [13] that has the potential to influence cognition when clinicians consciously or unconsciously draw on racial/ethnics stereotypes in clinical decision-making [10, 12].

Two recent systematic reviews summarise the literature on healthcare provider racism (37 studies, excluding students) [8] and the relationship between healthcare provider implicit racial/ethnic bias and health outcomes (15 studies, including students) [2]. These showed that racial/ethnic bias against non-white populations is common among health professionals with some evidence that this may negatively impact on health care encounters [2, 8].

Studies have employed a range of tools to measure different aspects of healthcare provider racial/ethnic bias. This includes direct measurement of explicit racial/ethnic bias, for example using questions asking about race preference, measurement of implicit racial/ethnic bias most commonly using the Implicit Association Test (IAT), and assessment of discrimination using tools such as clinical scenarios or observed patient encounters [2, 8].

A few studies have specifically examined racial/ethnic bias among medical students [14–17]. Researchers have highlighted the importance of identifying factors within medical education and training environments, such as curriculum content and training experiences, that may mitigate the effects of provider racial/ethnic bias on future clinical interactions and decision-making [9]. There may be opportunities to intervene in medical education and training to raise awareness of the potential impacts of racial/ethnic bias on health and develop strategies to minimise these for future health professionals. Better understanding of medical student racial/ethnic bias might also support improved learning and teaching environments.

### Study context

Although studies have been undertaken in this area internationally, we are not aware of any such studies with New Zealand medical student populations. New Zealand medical students live within a broader social context and will be exposed to a variety of narratives about Māori, both within and outside of medical school. This will include exposure to racialised and stereotypical beliefs and attitudes about Māori, evidence of which has been documented in university settings, the media and elsewhere [18–20]. This paper outlines the development of the *Bias and Decision-Making in Medicine (BDMM)* study that aims to investigate racial/ethnic bias in relation to Māori and New Zealand European^1^ people among New Zealand medical students and whether any such bias is related to clinical decision-making. BDMM is a subproject of the broader *Educating for Equity (E4E)* project involving Australia, Canada and New Zealand that seeks to “contribute to improving health professionals’ knowledge, attitudes and behaviours, plus share experiences and approaches to indigenous health teaching and learning in the area of chronic disease” [21].

It was important to undertake a comprehensive process of development and testing prior to the roll-out of the BDMM study to ensure its appropriateness for use within the New Zealand context and to optimise data quality [22]. This paper outlines the process that was used to develop, pretest and pilot the final web-based study content. It also aims to contribute to the limited detailed information published on the systematic development of similar studies internationally.

## Methods

**Fig. 1:**
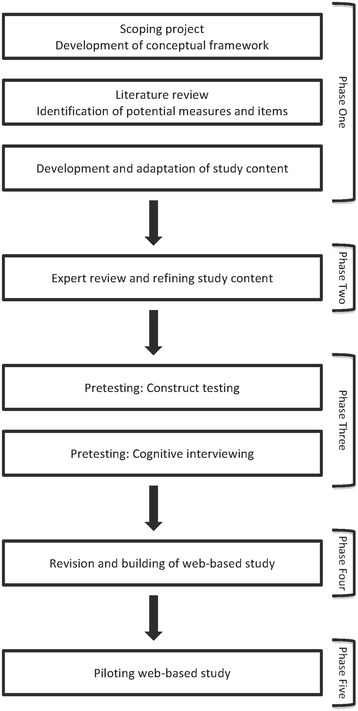
Overview of phases of development, testing and finalisation of study content

Study content was developed and pretested over eight stages within five phases (Fig. 1), drawing on established techniques for developing reliable and valid measurement tools [23, 24]. Ethics approval for study development and pretesting (Reference 010898) and piloting (Reference 011693) was granted by the University of Auckland Human Participants Ethics Committee and ratified by the University of Otago Human Ethics Committee.

### Phase one

#### Stage one: Scoping project and development of conceptual framework

This stage involved review of the literature on assessment of health professional racial/ethnic bias and the measurement of racial/ethnic bias towards indigenous peoples in general, and Māori specifically. The conceptual framework was developed in parallel to guide decisions about the inclusion of specific measures and items into the web-based study. The framework was informed by relevant literature, particularly the work of van Ryn and colleagues [9, 11] and Dovidio and colleagues [10, 25]. It defined the constructs intended to be measured as part of the overarching construct of racial/ethnic bias, as well as specifying specific domains of interest within each of the constructs. Specifically, it focused on dimensions of racial/ethnic bias identified in the work of van Ryn et al [9], p201, namely beliefs, attitudes, feelings and behaviours towards individuals or groups in respect of their race/ethnicity. Drawing on the work of Dovidio et al. [25] in relation to “prejudice, stereotyping and discrimination”, these dimensions were also conceptualised as capturing “cognitive”, “affective”, and “conative” (or behavioural) aspects of racial/ethnic bias [25]. Prejudice was approached as a construct that overlapped with both beliefs and feelings/evaluations [25]. Within each of the constructs, specific domains of interest for the BDMM study were outlined (see Table 1). For this study, individual-level racial/ethnic biases were understood as reflecting racialised ideologies, histories, and practices at a societal level [5, 9] and could manifest in overt, explicit ways, as well as in more subtle, implicit forms [9, 10].

**Table 1:** Overview of conceptual framework (study constructs and domains)

Racial/ethnic bias			
Construct	Domains of interest		Proposed study components
Beliefs [9, 25, 60]	Beliefs and stereotypes about Māori and New Zealand Europeans in general and Māori health in particular	General stereotypes - competence	*Explicit*: direct questions about Māori and New Zealand Europeans
			*Vignette*: questions about competence of hypothetical patient
		Health specific stereotypes - compliance	*Implicit*: Race and compliant patient IAT
			*Explicit*: direct questions about Māori and New Zealand European patients
			*Vignette*: questions about compliance of hypothetical patient
		Beliefs or knowledge about causes of ethnic inequalities in health between Māori and NZ European	*Explicit*: direct questions about Māori health, health outcomes and healthcare disparities
Evaluations (feelings) [9, 61, 62]	Feelings, judgements and expectations of Māori and NZ Europeans in general and patients in particular	General race/ethnic preference	*Implicit*: General race preference IAT
			*Explicit*: direct questions about preference for Māori relative to New Zealand European
		Warmth towards ethnic groups	*Explicit*: direct questions about warmth for Māori and New Zealand European ethnic groups
			*Vignette*: question about comfort with hypothetical patient
Discrimination (behaviours) [25, 60]	Behaviour towards and unfair treatment in clinical decision-making between Māori and NZ European	Diagnosis, management and treatment decision-making	*Vignette*: questions about decision-making for hypothetical patient
		Intent/motivation to treat	*Vignette*: question about intention to treat hypothetical patient
		Capacity to benefit	*Vignette*: question about perceived capacity of hypothetical patient to benefit

Note: Prejudice (attitude) draws on both beliefs (cognitive component) and evaluations (affective component) and is measured under these domains [25]

We identified three main study components from the literature to measure manifestations of racial/ethnic bias across the broad constructs outlined above (Table 1). These were assessments of: (1) *implicit* racial/ethnic bias using New Zealand-specific Implicit Association Tests (IATs); (2) *explicit* racial/ethnic bias using questions about medical student feelings, attitudes and beliefs; and, (3) clinical decision-making, using chronic disease vignettes.

The IAT is a response latency measure [26] that requires participants to rapidly sort stimuli representing four different concepts [27], and measures the “relative strength of association between pairs of concepts” [27], p62. It was the most commonly used implicit measure in studies of physician racial/ethnic bias [8], and was included to assess implicit feelings, beliefs and stereotype dimensions of racial/ethnic bias.

Questions asking directly about medical student feelings, attitudes and beliefs regarding Māori (relative to NZ European), Māori patients, and Māori health were included to assess warmth, preference, and cognitive domains of explicit racial/ethnic bias.

Clinical vignettes were identified as having the potential to measure both discrimination dimensions of bias in terms of patient diagnosis and management, as well as other dimensions of bias such as stereotypes/beliefs and affect toward individual hypothetical patients. Clinical vignettes have been used in similar studies to indirectly measure racial/ethnic bias by healthcare providers [8].

#### Stage two: Literature review and identification of potential measures and items

Following discussion and review of the framework by the research team, the literature was revisited to identify the specific instruments, measures and items for potential use across the three study components. An item-pool of potential measures and instruments was created in an Excel spreadsheet with information on: the construct, domain and sub-domains being measured; measure type; item wording; response options; item source; studies used in; and, use of measure previously in New Zealand and/or with medical students. A summarised version of the item pool was produced and reviewed by the research team to reach agreement on domains to assess within each of the broader constructs and prioritise potential items within each domain and construct. This also included consideration of response formats and how items may be scored or combined for analysis. Measures previously validated or used in other similar studies were prioritised where possible, for comparability of findings.

#### Stage three: Development and adaptation of study content

Identified potential content was adapted as required for use in a New Zealand context. This included changing the racial/ethnic group labels in United States (US) scales to equivalent ethnic group labels appropriate in the New Zealand context, as well as development of localised versions of the IAT, adaptation of clinical vignettes, and development of a set of statements to assess knowledge and beliefs about Māori health and inequities.

##### Implicit Association Test development

An ethnicity preference IAT and an ethnicity and compliant patient IAT were prioritised for inclusion in this study. These are based on the corresponding race preference IAT and race and compliant patient IAT, both developed for use in the US using images drawn from US populations [28, 29]. As the particular forms of the IAT we wished to include in our study had not been previously developed for use locally, adaptation required the sourcing and testing of appropriate stimuli (images and words) for the New Zealand context.

Prototypical Māori and European faces for use as images in the IATs were sourced by recruiting volunteers from talent agencies. Models were fully informed of the study purpose, paid for their image and consent for the use of the images in pretesting, piloting and the final study obtained. Headshot photographs were taken of 16 models under standardised conditions, with matching by other appearance factors (e.g. apparent age, gender, weight, hair length and wearing of glasses). Twenty-two images were selected for construct testing. Potential word stimuli for the ethnicity preference IAT were derived from corresponding Project Implicit [30] and Inquisit [31] race preference IATs. For the ethnicity and compliant patient IAT, word stimuli were derived from Sabin et al. [29] with additional researcher developed words (Table 2).

**Table 2:** Items included in construct testing

Study question/Area	Intended construct/concept	Items	Source/s	Testing
Patient surnames for use in clinical vignettes	Common NZ European/English language surname (corresponding to Māori surnames below)	Roberts Thomas Edwards James Williams Stephens	Researcher developed drawing on Department of Internal Affairs common surnames [63]	Unstructured sorting of NZ European surnames with Māori surnames AND Rating task
	Māori surname (transliterations of English language surnames above)	Ropata Tāmati Eruera Hemi Wiremu Tipene		
Photographs for use in IATs (ethnicity preference and ethnicity and compliant patient IAT)	Prototypical Māori images	7 headshots of women 3 of men	Actors/models	Unstructured sorting of Māori and NZ European images AND Rating task
	Prototypical NZ European images	8 headshots of women 4 men		
Word stimuli for ethnicity preference IAT	Good words	Set 1: Joy, Love, Peace, Wonderful, Pleasure, Glorious, Laughter, Happy	Set 1: Project implicit race preference IAT [30]	Unstructured sorting of corresponding ‘Good’ and ‘Bad’ word sets AND Rating task
		Set 2: Pleasure, Superb, Lovely, Wonderful, Marvelous, Glorious, Joyful, Beautiful	Set 2: Inquisit race preference IAT [31]	
	Bad words	Set 1: Agony, Terrible, Horrible, Nasty, Evil, Awful, Failure, Hurt	Set 1: Project implicit race preference IAT [30]	
		Set 2: Agony, Awful, Nasty, Horrible, Painful, Tragic, Humiliate, Terrible	Set 2: Inquisit race preference IAT [31]	
Word stimuli for ethnicity and compliant patient IAT	Compliant patient words	Willing, Cooperative, Compliant, Reliable, Adherent, Helpful, Motivated, Responsible, Trustworthy	[29] Researcher developed alternative options	Unstructured sorting of Compliant patient and Reluctant patient words AND Rating task
	Reluctant patient words	Reluctant, Doubting, Hesitant, Apathetic, Resistant, Lax, Averse, Slack, Opposed	[29] Researcher developed alternative options	
Words for use in explicit bias questions and vignette question	Competence	Intelligent Confident	Explicit bias questions on patient competence (adapted from [64]).	Rating task only
		Understands information	CVD vignette question asking likelihood of the patient understanding information [44]	

##### Vignette development

Cardiovascular disease and depression were identified as vignette topic areas by the research team, as both are chronic conditions with evidence of differences in healthcare by ethnicity in New Zealand [32–36] and where health-related ethnic stereotypes may be elicited. Clinical vignettes identified through literature review were discussed and prioritised by the research team. Considerations included developing clinically realistic scenarios that students would encounter, ensuring relevance to the study research questions, and inclusion of other factors within vignettes to control for potential biases [8, 37, 38].

Two clinical vignettes were subsequently selected and adapted for the New Zealand medical student context, with permission from original sources [39, 40]. The patient ethnicity in each vignette was signalled by describing the patient as New Zealand European or Māori and using common English language or corresponding Māori language surnames throughout the vignettes as additional ‘markers’ of ethnicity. The choice of names was finalised following construct testing (see below).

Vignette questions were selected in relation to the study’s aims to measure the constructs described above, and to capture both clinical decision-making as well as feelings and beliefs about the patients. The questions drew on examples used in other studies [14, 39, 41–44].

### Phase two

#### Stage four: expert review and refining study content

In a formal expert review process [45], four reviewers (with a range of expertise in Māori health, racism as a health determinant, psychology and quantitative tool development) reviewed the study concepts and overall study content. Reviewers were provided with a content guide containing a study overview, construct definitions, and an item pool. Reviewer guidelines and instructions asked them to comment on the conceptual basis of the study, individual items and response formats in terms of relevance, clarity, and appropriateness, and any perceived gaps [45]. Advice was also sought on prioritisation (where there were multiple items for the same measure) and need for pretesting. Following expert review, tools were modified and those requiring formal pretesting or additional review identified.

##### Clinical review of vignettes

To ensure scenarios were realistic, clinically sound and at the right level for final year medical students, clinical peer review of the vignette text and questions was undertaken [37, 38]. Five clinicians with expertise in medical education reviewed both vignettes and their associated questions. In addition, two cardiologists and two psychiatrists reviewed the cardiovascular and mental health vignettes respectively. Vignettes were revised accordingly.

### Phase three: Pretesting

Pretesting was undertaken to identify possible problems with questions and content [46, 47] and to provide information on the relevance and clarity of study items [48]. Pretesting focused on content that was newly developed or adapted for use in New Zealand, or not previously used or tested in New Zealand, or where there was uncertainty around the preferred option for inclusion. All pretesting participants were New Zealand university staff or students recruited via general email invitations. Eligibility was restricted to those aged over 18 years, and proficient in English. To minimise contamination of the final study sample, this group was limited to people without a close, personal relationship with a current medical student. A voucher was offered for participating in pretesting.

#### Stage five: Construct testing

Construct testing was carried out in May 2014 to assess alignment of selected study items with the intended underlying constructs [49, 50]. It incorporated both unstructured sorting [50, 51] and rating tasks [48, 49], undertaken while participants (*n* = 6) were observed by a researcher. Construct testing items included images and word stimuli for the IATs, surnames for inclusion in the clinical vignettes, and particular words used in some questions (summarised in Table 2).

For the unstructured sorting task, participants were given sets of items and asked to sort them into “like” groups, or groups of items they thought were similar. Additional prompts were used as necessary to elicit different ways items could be sorted. The researchers were interested in whether content was sorted into intended ‘target categories’ (aligned with intended underlying constructs) (Table 2).

For the rating tasks, participants were given a definition or explanation of a concept/construct, and a set of items relating to that concept/construct. For example, for the ‘Māori surname’ concept, the definition given was, ‘A surname or last name that sounds Māori or that you think people would generally think of as a Māori surname’. Participants were asked to rate how well they thought each item (photograph, word, or statement) “fits” with the definition provided, indicating for each item whether or not they thought it was a: ‘high’ fit, ‘moderate’ fit, ‘low’ fit, or unsure [49]. The items from the unstructured sorting were included again in the rating task alongside additional items relating to the concept of ‘competence’ (Table 2).

#### Stage six: Cognitive interviewing

Cognitive interviews were undertaken following the construct testing to assess how respondents understood and felt about the study content [52] and its appropriateness for use in New Zealand. The cognitive interviews focused on selected study content, namely questions about explicit racial/ethnic bias, questions that had been adapted or developed by the research team, and questions identified during the review process as potentially problematic or likely to benefit from additional testing [46]. Previously validated questions, or those used extensively in New Zealand, were not included in this phase. The specific items included in the cognitive interview questionnaire are outlined in Table 3.

**Table 3:** Items tested and summary of categories identified in cognitive interviews

Items tested	Source	Categories identified in cognitive interviews*
*1. Are you:* ▪ Male ▪ Female ▪ Other: ____	Statistics New Zealand Population Census 2013 question (adapted)	Understanding Response formatting Emotion
*2a. How would you best describe your socioeconomic background growing up?* ▪ Lower socioeconomic level ▪ Lower middle socioeconomic level/working class ▪ Middle socioeconomic level ▪ Upper-middle socioeconomic level ▪ Upper socioeconomic level	[39]	Understanding Task performance Response formatting
*2b. Would you describe your family income as: (mark one)* ▪ Low income ▪ Middle income ▪ Upper-Middle income ▪ High income	[65]	Understanding Task performance Response formatting
3. *Listed below are a few statements about your relationships with others. How much is each statement TRUE or FALSE for you?* 1. I am always courteous even to people who are disagreeable. 2. There have been occasions when I took advantage of someone. 3. I sometimes try to get even rather than forgive and forget. 4. I sometimes feel resentful when I don’t get my way. 5. No matter who I’m talking to, I’m always a good listener.	RAND 5-item Social Desirability Response [66]	Understanding Task performance Response formatting Emotion
5 response options (1. Definitely True; 2. Mostly True; 3. Don’t Know; 4. Mostly False; 5. Definitely False)		
4. *Which of the following best describes you in general?* ▪ I strongly prefer NZ Europeans to Māori ▪ I moderately prefer NZ Europeans to Māori ▪ I slightly prefer NZ Europeans to Māori ▪ I like NZ Europeans and Māori equally ▪ I slightly prefer Māori to NZ Europeans ▪ I moderately prefer Māori to NZ Europeans ▪ I strongly prefer Māori to NZ Europeans	[39] (adapted)	Understanding Response formatting Emotion
*5. Please rate your feelings of * *WARMTH * *toward the following groups using the “feeling thermometer scale” for each group.* 7-pt scale from LEAST WARM to MOST warm for each group separately (i.e. NZ Europeans, Māori)	[67] (adapted)	Understanding Task performance Response formatting Emotion
*6. Please answer the following questions by selecting your response on the scale below.* a. *In general, how* *competent* *do you think Māori/NZ European patients are?* b. *In general, how* *intelligent* *do you think Māori/NZ European patients are?* c. *In general, how* *confident* *do you think Māori/NZ European patients are?*	[64] (adapted)	Understanding Task performance Response formatting Emotion
Asked for each ethnic group separately; 7-pt scale from 1. Not at all [adjective] to 7. Extremely [adjective]		
*7. Please answer the following questions by selecting your response on the scale below.* a. *In general, how* *compliant* *do you think Māori/NZ European patients are?* b. *In general, how* *reliable* *do you think Māori/NZ European patients are?* c. *In general, how* *motivated* *do you think Māori/NZ European patients are?*	Developed by authors drawing on [64] and [68]	Understanding Response formatting Emotion
Asked each ethnic group separately; 7-pt scale from 1. Not at all [adjective] to 7. Extremely [adjective]		
*8. Major inequalities in health exist between Māori and NZ European in New Zealand. Please indicate how much you AGREE or DISAGREE with the following statements about Māori health and ethnic inequalities.* a) *Māori have a higher prevalence of chronic disease because they are genetically predisposed.* b) *Māori have worse health than NZ Europeans because of individual behavioural risk factors such as smoking and diet.* c) *Māori have worse health than NZ Europeans because of lower socioeconomic position.* d) *Māori have worse health than NZ Europeans because of racism in New Zealand.* e) *Māori have worse health than NZ Europeans because they present later for care.* f) *Health provider biases about patients based on their ethnicity contribute to poorer quality of care for Māori.* * g) If Māori patients receive less care than NZ European patients it is most likely due to their own preferences.*	Developed by authors	Understanding Response formatting Emotion
Likert scale from 1. Strongly disagree to 7. Strongly agree		

Note: *Category was identified by at least one participant in the cognitive interviews, Underlined and capitalised words are how they appear in the cognitive interview format

Nine participants took part in semi-structured cognitive interviews using think-aloud/read-aloud and probing techniques to elicit their thoughts on the questions and response options [47, 48, 52]. Participants were provided with a paper-based questionnaire, with the order of items based on the intended order for the final study. Participants were not asked to disclose their actual answers to the questions being tested [48]. At the end, the interviewer asked some questions on general thoughts about the overall study content. Participants were also asked to complete a brief demographic questionnaire, with questions on age, gender, ethnicity, highest education qualification, and clinical background.

Each interview was undertaken and audio-recorded by one of two senior researchers, who also took observational notes. Interviews were transcribed verbatim, imported into NVivo (version 10.0.4) and analysed by question to identify the range and types of problems [48], using a framework adapted from the literature [46, 52]. Coding focused on the following categories: understanding; task performance; and, response options. An additional category for emotional responses was added to capture information on participants’ levels of comfort with questions and other emotional responses.

Following the coding process in NVivo, codes and summaries were compared by item, with the two researchers producing an overall item summary by consensus. Recommendations for the revision of questions were made accordingly, then discussed and finalised with the research team.

### Phase four

#### Stage seven: Revision and building of web-based study

Following pretesting, revisions were made to study content and the final content and question order confirmed by the research team. The online questionnaire was built using Qualtrics (Provo, UT). As far as possible, the questionnaire was drafted to retain the formatting used in cognitive interviews. The online questionnaire was designed to require a common password for access. The participant information sheet and consent form were included as the first pages of the questionnaire.

The study content was built in four main modules with the order designed to minimise social desirability bias [29]. These were: 1) basic demographic questions; 2) the clinical vignettes; 3) two IATs; and, 4) social desirability, explicit ethnic bias and additional demographic questions. In the vignette module, the order and ethnicity of the patient in each vignette (Māori or NZ European) was programmed to be randomly assigned to participants (to balance combination and order effects, this was done in blocks of eight, the number of possible combinations).

In module 3, participants were asked to link out to the Project Implicit website to complete the IATs. These were built by Project Implicit using stimuli (images and words) supplied by the authors, and hosted on a separate website on Harvard servers. On completion of the IATs, participants were directed back to Qualtrics to complete the questionnaire (module 4). Participants could return to the Qualtrics questionnaire at any time with or without completing the IATs. Automatically generated embedded unique codes linked questionnaire and IAT responses anonymously.

Upon completion of the questionnaire, participants were provided with further information about the study and given the option of linking out to a separate website to enter their details to receive a voucher. These details could not be linked back to their questionnaire responses.

Extensive testing of the web-based study was undertaken by the research team prior to pilot testing. This included: testing on different operating systems, browsers, and devices (e.g. desktop, laptop, and mobile devices); proof-reading; checking variable coding; and, testing questionnaire logic.

### Phase five

#### Stage eight: Piloting web-based study

The complete web-based questionnaire was pilot tested to identify any remaining problems [24, 53], using a protocol as close as possible to the final study. Specifically, we wanted to test: 1) the time taken to do the questionnaire; 2) any remaining issues with study responses such as non-response to particular questions; 3) the distribution of responses; 4) technical functional issues of data collection and outputs, including checking the randomisation of vignettes, Qualtrics outputs and IAT functioning (e.g. the generation of a linking identifier, outputs and missing data); and, 5) any other issues raised in responses to the pilot specific questions.

The pilot sample was purposive. Participants were identified by the research team from staff within departments at the University of Auckland and the University of Otago (Wellington and Christchurch campuses) who had appropriate clinical knowledge to participate in the study.

In October 2014, 44 people were sent email invitations that included a link to the web-based questionnaire and a password. Participants were eligible if they were proficient in English, had trained as medical practitioners, and did not have a close personal relationship with a current medical student. Participation was voluntary and anonymous. Participants provided informed consent at the beginning of the web-based study and were offered a $20 voucher for participating. A reminder email was sent two days after the initial invitation.

The questionnaire included the same items chosen for the final study (with questions on medical school currently attending and training year removed) plus additional questions about doing the study for the pilot only (Table 4).

**Table 4:** Overview of pilot study questionnaire

Module order	Items/questions
Basic demographic questions	Age Gender Ethnicity
Vignettes	CVD Mental Health - patient ethnicity and vignette order were randomly assigned to participants in blocks of eight i.e. randomisation was evenly distributed for every eight participants - questions related to each vignette on patient diagnosis, management and participant perspectives of patients
Implicit bias	Ethnicity preference IAT Ethnicity and compliant patient IAT
Social desirability	The RAND 5-item Socially Desirable Response Set [66]
Explicit bias	Questions on explicit bias (knowledge of inequalities, compliance, competence, warmth, ethnicity preference)
Demographic questions	SES growing up Born in NZ Time in NZ (if not born here)
Pilot only questions	How long did it take you to complete the survey? Where did you complete the survey? Did you have any problem logging in to the survey? Did you have any technical difficulties undertaking this survey? Did you have any difficulties understanding and following the survey instructions? Do you have any further comments about how you found the process of completing the web-based survey?

## Results

### Construct testing results

In the unstructured sorting task, all six participants sorted surnames and the two sets of ethnicity preference IAT ‘good’ and ‘bad’ words into groups that aligned with the intended target categories. All participants sorted female images, and five of six people sorted male images, into ethnic group categories that aligned with intended target categories. Ethnic categories were not always the first sort, with images also sorted by age and hair type by some participants. Where images were sorted by ethnicity, a discrepancy with the intended category occurred seven times out of 122 sorts, for five images. The 18 words tested for use in the ethnicity and compliant patient IAT as representing the compliant and reluctant patient were sorted in line with intended categories by five participants. A discrepancy with the intended category occurred 12 times out of 90 sorts, for 9 words.

In the rating tasks, items were generally rated as high or moderate in relation to the provided construct, although ratings for ‘compliant patient’ and ‘reluctant patient’ word sets were more variable (see Additional file 1 for details of rating results).

Based on construct testing findings, two pairs of surnames were selected for use in the clinical vignettes (Williams/Wiremu and Stephens/Tipene). Twelve images for inclusion in the IATs were selected based on matched age, gender and prototypicality for Māori or NZ European appearing men and women (3 Māori men, 3 NZ European men, 3 Māori women and 3 NZ European women). The word sets from either race preference IAT were deemed suitable for inclusion in our ethnicity preference IAT. As Project Implicit was contracted to build the IAT for the study, their race preference IAT wordsets were prioritised for inclusion. Words and phrases intended to align with the construct of competence were also rated as suitable. The compliant patient words from the race and compliant patient IAT source were deemed satisfactory although some changes were made for the reluctant patient words. This involved replacing ‘lax’ with ‘slack’ as a more commonly used term in New Zealand, with ‘slack’ testing better than ‘lax’, and replacing ‘doubting’ with ‘averse’ as ‘averse’ scored higher in the rating task.

### Cognitive interview results

Of the nine participants, most were aged between 25–34 years (*n* = 5), were female (*n* = 6), identified with a European ethnic group (*n* = 6), had a tertiary qualification (*n* = 8), and reported having clinical experience (*n* = 7). Table 3 includes summarised findings from the cognitive interviews in terms of the four categories of analysis: understanding, task performance, response options, and emotional responses.

The main outcomes from the cognitive interview phase were changes to the proposed ordering of questions, and wording changes to improve clarity and coherence of some items. Specifically, this included changes to question order in the explicit bias module based on participant feedback about potential discomfort with answering questions on ethnic preference (Item 4) or comparing ethnic groups in relation to competence and compliance (Items 6 and 7). Although participants understood the value of these questions, they were reordered to the end of the module. The competence and compliance questions (Items 6 and 7) were re-ordered so that the questions were asked firstly in reference to NZ European patients, then Māori patients. All questions were made optional for the final study, with participants able to proceed through the questionnaire without being required to provide an answer. The explicit bias module was also reordered to improve its flow and coherence; this put questions about health contexts (Item 8) and patient groups (Items 6 and 7) at the start, followed by more general questions about ethnic groups (Items 4 and 5).

The gender item was retained, with the ‘write-in’ text box for those who ticked ‘Other’ made optional. There was no clear preference between the two socioeconomic background questions tested (Items 2a and 2b), although issues with the reference timeframe and response categories for both questions were identified. Therefore, Item 2a was chosen, with the words ‘growing up’ added to the question stem to clarify that the question referred to participants’ socioeconomic background. The term ‘competent’ was changed to ‘capable’ in Item 6. Minor wording changes were made to the introduction to the social desirability question (Item 3), as well as to the question stem and some of the statements in the belief statements set (Item 8). One additional statement was also included to capture beliefs about the role of the health system (i.e. “Māori have worse health than NZ Europeans because the health system does not deliver equitable care to Māori”), as two participants felt that the current item set did not cover this area sufficiently.

### Pilot testing results

Eighteen participants undertook the pilot study, although one had a technical issue and did not proceed past the IATs because of a slow connection. Of the 17 people who completed the study, all were aged 30+, with 11 women and 6 men. Using prioritised ethnicity [54], 2 identified as Māori, 2 as Other and 13 as NZ European.

The consent process functioned as required. The average self-reported time to complete the study was between 20–30 min. Based on the survey software, the average was 27 min (excluding one outlier of 4 h).

The randomisation of clinical vignettes and outputs from Qualtrics functioned well, as did the generation of anonymous identifiers to link Qualtrics questionnaire data to Project Implicit held IAT data.

Some problems were identified with the IAT module. Two participants had no IAT data and no unique linking identifier, indicating they did not link to the IAT website. Five participants linked to the IATs (a linkage ID had been generated) but had missing or incomplete data. Feedback from participants included that the button to link to the IATs was quite small and could be missed, and that the IATs required a lot of concentration.

For all other questions, there was very little missing data and no obvious ceiling or floor effects. Results from the pilot specific questions showed that 11 people logged into the study from their workplace and 6 from home, with no problems identified with location. One person reported a problem logging into the study as they did not realise two boxes needed checking to proceed.

Overall, the pilot was well received aside from the few minor issues outlined above. Findings were reviewed by the research team, and the study content and format finalised. Technical problems with the IAT were fed back to Project Implicit. A number of minor changes were made to the web-based study to mitigate problems identified in the pilot. These included: adding additional information to the participant invitation regarding using a computer with a keyboard (as the IAT was not set up for use with a touch device); the need to concentrate during the study and the time it will take; and making the consent process more prominent to avoid confusion over the need to tick two boxes. Changes were also made to improve transition to the IATs. The link to the IATs was made more prominent to minimise the risk of missing this; the heading size on each IAT task was increased so participants could more clearly see where the second IAT started; and, a back button was included on the questionnaire page following the IAT link to allow participants to return to the IAT page if they accidentally skipped it.

#### Final questionnaire

The final questionnaire content can be found in Additional file 2. Each main module can be used to assess aspects of racial/ethnic bias amongst medical students. Differential responding to the clinical scenarios (including differential clinical decision making by randomised patient ethnicity) can be assessed by comparing response profiles to each question (e.g. mean scores on questions). The IAT component can be scored using standard guidelines to produce D scores that give a measure of implicit bias (in this case bias for NZ European relative to Māori) [55]. For the explicit bias module, frequencies of responses in particular categories and/or means can be calculated to assess ethnic preference and bias in knowledge and beliefs about ethnic health inequalities between NZ European and Māori. For items examining warmth, competence, and compliance, explicit bias can be examined using mean paired differences (as respondents give answers for both ethnic groups). Total competence and compliance scores can be calculated by summing the responses to each of the three individual items in each scale. Results from each of these modules can be used to explore how implicit and explicit bias are related to each other, and how these biases are associated with clinical decision-making and evaluations of individual patients.

## Discussion

The development, pretesting and piloting process for this study provided important and useful information that helped to prioritise and refine the final content and delivery process. Undertaking a systematic and in-depth study development process is important in order to maximise data quality, including validity, with the inclusion of measures that capture the intended underlying constructs in a reliable and appropriate manner [23, 24]. In particular, the articulation of an underlying conceptual framework guided decisions about the inclusion of specific measures and potential items into the web-based study. Expert review helped ensure clarity of concepts and item relevance as well as informing prioritisation of items for pretesting. Clinical review provided useful information about whether clinical scenarios were realistic, clinically sound and at the appropriate level for the intended target population (final year medical students).

The use of multiple pretesting methods allowed us to test different aspects of the study content [46–49, 51, 52]. For example, sorting and rating tasks in the construct testing phase assisted in prioritising study materials that best aligned with the intended underlying concepts and constructs being measured, such as prototypical images of Māori and NZ European faces. This was particularly important given that some of the measures had not previously been used in New Zealand and the nature of the measures meant that there was limited ability to generalise from their use internationally. Cognitive interviews provided valuable information on how participants understood and responded to questions as well as how they felt about particular questions. This helped to inform the order of questions to improve question flow and better contextualise questions within a healthcare setting as well as minimise any potential distress and dropout. Finally, piloting of the study to assess the functionality of the delivery process and responses to study items was successful, resulting in a few minor changes intended to improve clarity and data completeness.

Reviews of health provider racism and ethnic bias studies have noted the need for more conceptual clarity [2], the strength in using different types of measures (both direct and indirect) that help mitigate the limits of each other [8] and the need to link measures of implicit and explicit bias to health care outcomes [2]. Our study explicitly addressed these issues in its development. The final content was designed to include measures that captured the major conceptual elements of ethnic bias (beliefs, feelings and evaluations, discrimination) among medical students and in relation to health care. The three main components of the study (chronic disease vignettes, implicit association tests, explicit ethnic bias questions) utilised different types of measures, allowing the ability to assess various manifestations and aspects of ethnic bias among medical students as well as how they inter-relate. For example, we wanted to be able to assess how implicit and explicit measures of ethnic bias relate to each other and to clinical decision-making and ethnic health inequities. Other research has used similar study designs and components among physicians [29, 39, 56–58]. A few studies have used more limited measures to examine racial/ethnic bias among medical students [15–17] and we are aware of only one other that has incorporated implicit and explicit bias measures with measures of clinical decision-making among medical students [14].

Similar development methods have been described in other studies of healthcare provider ethnic bias including the use of conceptual frameworks [59], expert review of vignettes [14, 56–58], independent assessment of prototypical photographs [39], and piloting of some study elements [14, 58]. However, these development methods are often only briefly described in final results papers with detailed information on the development of study design and content, pretesting and piloting not readily available in published form. Our study draws on previous research, with adaptation and development of measures for the New Zealand context allowing for both the comparison of findings and increased validity of measures.

We believe that the systematic approach to development and testing outlined here will provide useful information for those undertaking similar work, particularly where the adaptation and development of measures for use in other locations and contexts may be necessary. The process described for this study also provides important information on the provenance of items for potential use in other studies locally. For example, the series of questions on understandings of ethnic health inequities may be useful in other areas of health services research in New Zealand.

The development of the BDMM study was focussed on the New Zealand context and ensuring the content was most relevant for final year medical students. Therefore, some materials and content may not be relevant in other locations or for other groups. For example, patient names and IAT images are New Zealand specific, and in the case of clinical scenarios, these are aimed at the knowledge, skills and clinical experience levels of final year medical students. We were unable to involve the final year medical students in the study development and pretesting as the invitation to participate in the final study was intended to be sent to all final year medical students. However, we did try to invite participants with overlapping characteristics with medical students in pretesting and piloting i.e. other health students and health professionals. In addition, the number of participants in the pretesting phase was relatively small, although in line with other studies using similar methods [49, 51]. A separate final step in the development of the ethnic bias measures discussed here would be to examine their validity in a large sample of respondents. Such validation could include assessing concurrent validity between new and existing measures, and considering whether composite measure scores (e.g. overall measure of perceived competence for a given ethnic group) align with proposed underlying constructs of ethnic bias.

## Conclusions

In conclusion, this paper describes a systematic process to develop and produce a study to measure ethnic bias and clinical decision-making among final year medical students in New Zealand. The role of racial/ethnic bias among health providers in contributing to ethnic health inequity is an emerging area of research focus [4]. As such, it is important to develop robust and multifaceted research tools to support investigation in this field [8]. It is hoped that this paper has utility for other researchers working in this area by informing potential development processes and identifying possible measurement tools.

## Abbreviations

BDMM, Bias and Decision-Making in Medicine; E4E, Educating for Equity; IAT, Implicit association test; NZ, New Zealand; US, United States.

## Additional files

Additional file 1: Additional Table: Numbers of participants (n) rating items as high, moderate, low. Results from rating tasks in construct testing. (DOCX 29.5 KB) Additional file 2: BDMM Questionnaire. Final questionnaire developed for the main study. (DOCX 34 kb)

## Footnotes

1 New Zealand European is the numerically dominant ethnic group in New Zealand and the term used to describe this group in official statistics.
