# Supplementary material for: Development and testing of study tools and methods to examine ethnic bias and clinical decision-making among medical students in New Zealand: The Bias and Decision-Making in Medicine (BDMM) study
Source: BMC Med Educ. 2016 Jul 11;16:173. doi: 10.1186/s12909-016-0701-6 (PMC4940847; doi:10.1186/s12909-016-0701-6)
Supplement: Additional file 1: — Additional Table: Numbers of participants (n) rating items as high, moderate, low. Results from rating tasks in construct testing. (DOCX 29.5 KB) [file 12909_2016_701_MOESM1_ESM.docx]

***Additional Table: Numbers of participants (n) rating items as high, moderate, low***

| **Construct/concept** | **Item** | **High (n)** | **Moderate (n)** | **Low (n)** | **Missing/unsure (n)** |
| --- | --- | --- | --- | --- | --- |
| **Māori surnames** |  |  |  |  |  |
|  | Tipene | 4 | 1 | 1 |  |
|  | Tāmati | 4 | 1 | 1 |  |
|  | Wiremu | 4 | 1 | 1 |  |
|  | Ropata | 5 |  | 1 |  |
|  | Eruera | 3 | 1 | 1 | 1 |
|  | Hemi | 3 | 1 | 1 | 1 |
| **NZ European surnames** | |  |  |  |  |
|  | Roberts | 5 | 1 |  |  |
|  | Thomas | 4 | 1 | 1 |  |
|  | Edwards | 4 | 2 |  |  |
|  | James | 4 | 2 |  |  |
|  | Williams | 5 | 1 |  |  |
|  | Stephens | 5 | 1 |  |  |
| **Māori images** |  |  |  |  |  |
|  | Image 1 | 5 |  | 1 |  |
|  | Image 2 | 4 | 2 |  |  |
|  | Image 3 | 4 | 2 |  |  |
|  | Image 4 | 4 | 1 | 1 |  |
|  | Image 5 | 5 | 1 |  |  |
|  | Image 6 | 5 | 1 |  |  |
|  | Image 7 | 4 | 2 |  |  |
|  | Image 8 | 4 | 2 |  |  |
|  | Image 9 | 4 | 1 | 1 |  |
|  | Image 10 | 4 | 2 |  |  |
| **NZ European images** |  |  |  |  |  |
|  | Image 11 | 4 | 2 |  |  |
|  | Image 12 | 5 | 1 |  |  |
|  | Image 13 | 5 | 1 |  |  |
|  | Image 14 | 4 | 2 |  |  |
|  | Image 15 | 4 | 2 |  |  |
|  | Image 16 | 3 | 3 |  |  |
|  | Image 17 | 4 | 2 |  |  |
|  | Image 18 | 2 | 3 | 1 |  |
|  | Image 19 | 5 | 1 |  |  |
|  | Image 20 | 2 | 4 |  |  |
|  | Image 21 | 4 | 2 |  |  |
|  | Image 22 | 4 | 2 |  |  |
| **Good words** |  |  |  |  |  |
|  | Joy | 6 |  |  |  |
|  | Love | 6 |  |  |  |
|  | Peace | 6 |  |  |  |
|  | Wonderful | 5 | 1 |  |  |
|  | Pleasure | 4 | 2 |  |  |
|  | Glorious | 6 |  |  |  |
|  | Laughter | 5 | 1 |  |  |
|  | Happy | 5 | 1 |  |  |
|  | Superb | 6 |  |  |  |
|  | Lovely | 5 | 1 |  |  |
|  | Marvelous | 6 |  |  |  |
|  | Joyful | 5 | 1 |  |  |
|  | Beautiful | 6 |  |  |  |
| **Bad words** |  |  |  |  |  |
|  | Agony | 6 |  |  |  |
|  | Terrible | 5 | 1 |  |  |
|  | Horrible | 6 |  |  |  |
|  | Nasty | 6 |  |  |  |
|  | Evil | 6 |  |  |  |
|  | Awful | 5 | 1 |  |  |
|  | Failure | 6 |  |  |  |
|  | Hurt | 6 |  |  |  |
|  | Painful | 6 |  |  |  |
|  | Tragic | 6 |  |  |  |
|  | Humiliate | 6 |  |  |  |
| **Compliant patient words** | |  |  |  |  |
|  | Willing | 4 | 2 |  |  |
|  | Cooperative | 4 | 2 |  |  |
|  | Reliable | 5 | 1 |  |  |
|  | Adherent | 2 | 2 |  | 2 |
|  | Helpful | 2 | 3 | 1 |  |
|  | Motivated | 4 | 2 |  |  |
|  | Responsible | 4 | 2 |  |  |
|  | Trustworthy | 3 | 3 |  |  |
| **Reluctant patient words** | |  |  |  |  |
|  | Doubting | 4 |  | 2 |  |
|  | Hesitant | 2 | 4 |  |  |
|  | Apathetic | 2 | 2 |  | 2 |
|  | Resistant | 2 | 3 | 1 |  |
|  | Lax | 2 | 2 |  | 2 |
|  | Averse | 4 | 1 |  | 1 |
|  | Slack | 2 | 4 |  |  |
|  | Opposed | 4 |  | 2 |  |
| **Competence** |  |  |  |  |  |
|  | Intelligent | 2 | 4 |  |  |
|  | Confident | 2 | 3 | 1 |  |
|  | Understands information | 6 |  |  |  |
