# Supplementary material for: Development and testing of study tools and methods to examine ethnic bias and clinical decision-making among medical students in New Zealand: The Bias and Decision-Making in Medicine (BDMM) study
Source: BMC Med Educ. 2016 Jul 11;16:173. doi: 10.1186/s12909-016-0701-6 (PMC4940847; doi:10.1186/s12909-016-0701-6)
Supplement: Additional file 2: — BDMM Questionnaire. Final questionnaire developed for the main study. (DOCX 34 kb) [file 12909_2016_701_MOESM2_ESM.docx]

**Note: Please refer to the full manuscript for details on the sources of individual items**

**BDMM Questionnaire**

*Please only complete this survey once.   The following section will ask you some basic demographic questions. Then you will read two clinical vignettes and be asked a set of standard survey questions about the cases following each vignette.*

A1. Please indicate your age in years:   (choose age from the drop-down menu)

- 20
- 21
- 22
- 23
- 24
- 25
- 26
- 27
- 28
- 29
- 30+

A2. Are you:

- Male
- Female
- Other ____________________

A3. Which ethnic group do you belong to?       Mark the space or spaces that apply to you.

- NZ European
- Māori
- Samoan
- Cook Island Māori
- Tongan
- Niuean
- Chinese
- Indian
- Other (such as Dutch, Japanese, Tokelauan). Please state: ____________________

A4. Please indicate your current year of medical training:

- 6th year (Trainee Intern)
- 5th year

*Instructions: Please read the following case scenario carefully. Your task is to make decisions about the patient’s diagnosis and treatment prior to discussing your recommendations with your supervising consultant. You may not have all the information you feel you need to answer the questions. However, with the information you do have, please answer all of the following questions as best you can.*

Part 1 You are a first year house officer in a regional hospital emergency department. Mr Wiremu/Williams, a 50-year-old Māori/NZ European male school teacher, presents to the emergency department with chest pain. He appears to be in a lot of pain describing it as “sharp, like being stabbed with a knife” and pointing to the mid sternum. He has had it about three hours, and it has waxed and waned, but is now an 8 out of 10 in intensity. The pain is not exacerbated by movement or deep inspiration. It does not radiate and is not accompanied by SOB, nausea, or diaphoresis. He has a history of smoking and well-controlled hypertension, but no other risk factors for coronary artery disease. His vital signs, oxygen saturation, and physical exam are normal except for some mild sternal tenderness to palpation. His ECG shows 2 mm horizontal ST elevations in the anterior leads, but there is no prior ECG for comparison and there will be a delay until the lab is able to complete cardiac enzymes analysis. You do not have access to a cardiac catheterisation lab. He has no absolute contraindications to thrombolysis.

B1. Please assess the likelihood that Mr Wiremu's/Mr Williams’ pain is due to coronary artery disease:

- 1 very unlikely (<20%)
- 2 somewhat unlikely (20-40%)
- 3 as likely as not (41-59%)
- 4 somewhat likely (60-80%)
- 5 very likely (>80%)

B2. Using the information available, what would your recommendation be regarding thrombolysis for Mr Wiremu/Mr Williams when you discuss this case with your consultant?

- 1 would definitely recommend thrombolysis
- 2 would probably recommend thrombolysis
- 3 not sure
- 4 would probably NOT recommend thrombolysis
- 5 would definitely NOT recommend thrombolysis

B3. In your opinion, how effective is thrombolysis in the treatment of acute ST elevation myocardial infarction (STEMI) (assuming primary angioplasty is not available)?

- 1 very ineffective
- 2 somewhat ineffective
- 3 don’t know
- 4 somewhat effective
- 5 very effective

Part 2 You relay the history to a cardiologist over the phone, and he reviews an electronic copy of the ECG. After a thorough discussion, he advises you to give Mr Wiremu/Mr Williams thrombolysis since there are no absolute contraindications and he is almost certainly having an MI. You then speak with Mr Wiremu/Mr Williams about his current medical condition and the risks and benefits of thrombolysis. He is very anxious about thrombolysis and seems reluctant to agree to it.

B4. How would you rate the likelihood that Mr Wiremu/Mr Williams will ultimately refuse thrombolysis after further discussion?

- 1 very unlikely (<20%)
- 2 somewhat unlikely (20-40%)
- 3 as likely as not (41-59%)
- 4 somewhat likely (60-80%)
- 5 very likely (>80%)

B5. How would you rate the likelihood that Mr Wiremu/Mr Williams understands medical advice regarding thrombolysis?

- 1 very unlikely (<20%)
- 2 somewhat unlikely (20-40%)
- 3 as likely as not (41-59%)
- 4 somewhat likely (60-80%)
- 5 very likely (>80%)

B6. If Mr Wiremu/Mr Williams refuses thrombolysis, how would you describe your subsequent management regarding thrombolysis?

- 1 I would not try to persuade him any further
- 2
- 3
- 4
- 5 I would try very hard to persuade him

B7. How comfortable or uncomfortable would you feel working with Mr Wiremu/Mr Williams?

- 1 very uncomfortable
- 2 somewhat uncomfortable
- 3 neutral
- 4 somewhat comfortable
- 5 very comfortable

Part 1 You are a Year 6 Medical Student working in a General Practice and are seeing a patient prior to the patient being reviewed by the GP. Your patient, Mr Tipene/Mr Stephens, is a 44 year-old Māori/NZ European male electrician. He is being seen with a four-month history of muscle aches, insomnia, headache and irritability. He was in relatively good health prior to this. He has obtained intermittent relief from headache by using paracetamol and sometimes takes a multivitamin. He complains, “food just doesn’t taste good anymore”. He has been finding it harder lately to concentrate at work, and to get up the energy to socialise with friends and family. He wonders if he will ever feel normal again, yet denies any stress or significant problems in his life.   He has been a patient with this GP practice for 15 years but has had few appointments in that time. Clinical records indicate past presentations for acute migraine and tonsillitis. He was also briefly treated with an anti-depressant for “depression” aged 19 after a relationship breakup. He has been married for 21 years with two adult children. He does not smoke, and drinks 2 cups of coffee/day. He denies drinking alcohol or other substance use. Physical exam is unremarkable.

C1. How reliable do you think the information provided by this patient is?

- 1 very unreliable
- 2 somewhat unreliable
- 3 as reliable as not
- 4 somewhat reliable
- 5 very reliable

C2. Please assess the likelihood that Mr Tipene's/Mr Stephens’ symptoms are due to depression:

- 1 very unlikely (<20%)
- 2 somewhat unlikely (20-40%)
- 3 as likely as not (41-59%)
- 4 somewhat likely (60-80%)
- 5 very likely (>80%)

C3. Based on the information you have, how would you rate the severity of Mr Tipene's//Mr Stephens’ symptoms?

- None-slight
- Mild
- Moderate
- Severe

C4. What is the likelihood that Mr Tipene/Mr Stephens will form a good therapeutic alliance with his GP?

- 1 very unlikely
- 2 somewhat unlikely
- 3 as likely as not
- 4 somewhat likely
- 5 very likely

C5. What initial management approach would you recommend? Please rank the options in order of your most likely initial management approach (rank from 1=most likely to 5=least likely). Type your ranking in the boxes below.

______ a. Encourage self-help strategies (such as exercise, sleeping well, stress reduction, problem solving)

______ b. Refer to social support and counselling services

______ c. Recommend anti-depressant treatment

______ d. Refer to primary care brief intervention service (approximately 5 sessions of psychological therapy)

______ e. Commence anti-depressant and refer to specialist mental health services

C6. Please rate the likelihood that Mr Tipene/Mr Stephens will benefit from your selected initial management approach:

- 1 very unlikely
- 2 somewhat unlikely
- 3 as likely as not
- 4 somewhat likely
- 5 very likely

Part 2 You arrange to see Mr Tipene/Mr Stephens with his GP. The GP gathers additional information, which includes Mr Tipene/Mr Stephens reporting occasional thoughts of suicide. The GP subsequently considers that Mr Tipene/Mr Stephens is experiencing a recurrence of depression of moderate severity. The GP recommends commencing him on an anti-depressant and referral to specialist mental health services.

C7. How would you rate the likelihood that Mr Tipene/Mr Stephens will take his anti-depressant medication as prescribed?

- 1 very unlikely
- 2 somewhat unlikely
- 3 as likely as not
- 4 somewhat likely
- 5 very likely

C8. How would you rate the likelihood of Mr Tipene/Mr Stephens attending his appointment for assessment by specialist mental health services?

- 1 very unlikely
- 2 somewhat unlikely
- 3 as likely as not
- 4 somewhat likely
- 5 very likely

*You will now be asked to take 2 brief cognitive tests (Implicit Association Tests) that will ask you to quickly categorise a series of concepts. This will open in a new window. Click here to start the tests. You will return to this page after finishing the cognitive tests. At that point, click the button below to continue. Click the above button to go back if you missed the cognitive tests link.*

*Ethnicity preference IAT*

|  | Target categories | Attribute categories |
| --- | --- | --- |
| Labels | NZ European  Māori | Good  Bad |
| Stimuli | NZ European female 1  NZ European female 2  NZ European female 3  NZ European male 1  NZ European male 2  NZ European male 3  Māori female 1  Māori female 2  Māori female 3  Māori male 1  Māori male 2  Māori male 3 | Joy  Love  Peace  Wonderful  Pleasure  Glorious  Laughter  Happy  Agony  Terrible  Horrible  Nasty  Evil  Awful  Failure  Hurt |

*Ethnicity and compliant patient IAT*

|  | Target categories | Attribute categories |
| --- | --- | --- |
| Labels | NZ European patient  Māori patient | Compliant patient  Reluctant patient |
| Stimuli | NZ European female 1  NZ European female 2  NZ European female 3  NZ European male 1  NZ European male 2  NZ European male 3  Māori female 1  Māori female 2  Māori female 3  Māori male 1  Māori male 2  Māori male 3 | Willing  Cooperative  Compliant  Reliable  Adherent  Helpful  Reluctant  Hesitant  Apathetic  Resistant  Averse  Slack |

The following questions ask about your relationships in day-to-day life. Please remember, all your responses will be completely anonymous.

D1. Listed below are a few statements about your relationships with others. How much is each statement TRUE or FALSE for you?

a. I am always courteous even to people who are disagreeable.

- Definitely True
- Mostly True
- Don't Know
- Mostly False
- Definitely False

b. There have been occasions when I took advantage of someone.

- Definitely True
- Mostly True
- Don't Know
- Mostly False
- Definitely False

c. I sometimes try to get even rather than forgive and forget.

- Definitely True
- Mostly True
- Don't Know
- Mostly False
- Definitely False

d.  I sometimes feel resentful when I don’t get my way.

- Definitely True
- Mostly True
- Don't Know
- Mostly False
- Definitely False

e. No matter who I’m talking to, I’m always a good listener.

- Definitely True
- Mostly True
- Don't Know
- Mostly False
- Definitely False

The following section will ask you about your perspectives on ethnicity and health in New Zealand.

D2. Major inequalities in health exist between Māori and NZ European in New Zealand. Please indicate how much you AGREE or DISAGREE with the following statements about Māori health and ethnic inequalities.

a. The higher prevalence of chronic disease among Māori is because they are genetically predisposed.

- Strongly Disagree


- Strongly Agree

b. Māori have worse health than NZ Europeans because of individual behaviours such as smoking and diet.

- Strongly Disagree


- Strongly Agree

c. Māori have worse health than NZ Europeans because of lower socioeconomic position.

- Strongly Disagree


- Strongly Agree

d. Māori have worse health than NZ Europeans because of racism in New Zealand.

- Strongly Disagree


- Strongly Agree

e. Māori have worse health than NZ Europeans because they do not seek care early enough.

- Strongly Disagree


- Strongly Agree

f. Ethnic bias by health providers leads to poorer quality of care for Māori.

- Strongly Disagree


- Strongly Agree

g. If Māori patients receive less care than NZ European patients it is most likely due to Māori preferences for care.

- Strongly Disagree


- Strongly Agree

h. Māori have worse health than NZ Europeans because the health system does not deliver equitable care to Māori.

- Strongly Disagree


- Strongly Agree

D3. Please answer the following questions about patients by selecting your response on the scale below.

a. In general, how compliant do you think NZ European patients are?

- Not at all compliant


- Extremely compliant

b. In general, how reliable do you think NZ European patients are?

- Not at all reliable


- Extremely reliable

c. In general, how motivated do you think NZ European patients are?

- Not at all motivated


- Extremely motivated

d. In general, how compliant do you think Māori patients are?

- Not at all compliant


- Extremely compliant

e. In general, how reliable do you think Māori patients are?

- Not at all reliable


- Extremely reliable

f. In general, how motivated do you think Māori patients are?

- Not at all motivated


- Extremely motivated

D4. Please answer the following questions about patients by selecting your response on the scale below.

a. In general, how capable do you think NZ European patients are?

- Not at all capable


- Extremely capable

b. In general, how intelligent do you think NZ European patients are?

- Not at all intelligent


- Extremely intelligent

c. In general, how confident do you think NZ European patients are?

- Not at all confident


- Extremely confident

d. In general, how capable do you think Māori patients are?

- Not at all capable


- Extremely capable

e. In general, how intelligent do you think Māori patients are?

- Not at all intelligent


- Extremely intelligent

f. In general, how confident do you think Māori patients are?

- Not at all confident


- Extremely confident

D5. Please rate your feelings of WARMTH toward the following groups using the “feeling thermometer scale” for each group.

a. NZ Europeans

- Feel LEAST WARM Toward This Group

- Neutral

- Feel MOST WARM Toward This Group

b. Māori

- Feel LEAST WARM Toward This Group

- Neutral

- Feel MOST WARM Toward This Group

D6. Which of the following best describes you in general?

- I strongly prefer NZ Europeans to Māori
- I moderately prefer NZ Europeans to Māori
- I slightly prefer NZ Europeans to Māori
- I like NZ Europeans and Māori equally
- I slightly prefer Māori to NZ Europeans
- I moderately prefer Māori to NZ Europeans
- I strongly prefer Māori to NZ Europeans

D7. How would you best describe your socioeconomic background growing up?

- Low
- Lower-middle
- Middle
- Upper-middle
- High

D8: Please indicate the University you are enrolled at for your final year of medicine:

- University of Auckland
- University of Otago

D9. Were you born in New Zealand?

- Yes
- No

D10. If you live in New Zealand but were not born here, answer this question. When did you first arrive to live in New Zealand?

- Pre 1970
- 1970
- 1971
- 1972
- 1973
- 1974
- …. 2014
